# Time-restricted feeding relieves high temperature-induced impairment on meat quality by activating the Nrf2/HO-1 pathway, modification of muscle fiber composition, and enriching the polyunsaturated fatty acids in pigs

**DOI:** 10.1007/s44154-024-00182-w

**Published:** 2024-09-14

**Authors:** Zhaojian Li, Yiting Wang, Peng Yuan, Yanli Zhu, Ping Hu, Tongxing Song, Rui Liu, Hao-Yu Liu, Demin Cai

**Affiliations:** 1 Laboratory of Animal Physiology and Molecular Nutrition, Jiangsu Key Laboratory of Animal Genetic Breeding and Molecular Design, College of Animal Science and Technology, Yangzhou University, Yangzhou, 225009 China; 2 College of Animal Science and Technology, Huazhong Agricultural University, Wuhan, 430070 China

**Keywords:** Meat quality, Time-restricted feeding, High ambient temperature, Muscle fiber composition, Nrf2, Fatty acids composition

## Abstract

To assess the effects of a time-restricted feeding (TRF) regimen on meat quality of pigs exposed to high ambient temperature, a two-month feeding and heat treatment (HT) trial was conducted using a 2 × 2 factorial design. A total of 24 growing pigs (11.0 ± 1.9 kg) were randomly divided into four groups: thermal neutral group (NT, 24 ± 3 °C), HT group (exposed to a high temperature at 35 ± 2 °C from 11:00 to 15:00), TRF group and HT + TRF group (HT and TRF co-treatment group, *n* = 6 for each group). Pigs in TRF groups got access to feed within 5 h from 9:00 to14:00, while the others were fed at 6:00, 11:30, and 16:00. All pigs received the same diet during the trail. The results showed that HT increased the drip loss, shear force, lightness, and malondialdehyde production in *Longissimus thoracis et lumborum* (LTL) muscle. TRF reversely reduced the shear force and drip loss, accompanied by decreased intramuscular fat and increased moisture content. Enhanced fiber transformation from type 1 to type 2b and down-regulated expression of muscle growth-related genes were observed by HT, while TRF suppressed the fiber transformation and expression of muscle atrophy-related genes. Furthermore, TRF restored the diminished protein expressions of Nrf2 and HO-1 in LTL muscle by chronic HT. Accumulation of HSP70 in muscle of HT group was reduced by treatment of TRF. HT declined the expression of vital genes involved in fatty acids poly-desaturation and the proportion of (polyunsaturated fatty acids) PUFAs, mainly omega-6 in LTL muscle, while TRF group promoted the expression of poly-desaturation pathway and displayed the highest proportion of PUFAs. These results demonstrated that TRF relieved the chronic high temperature affected meat quality by the restored expression of Nrf2/HO-1 anti-oxidative cascade, modified muscle fiber composition, and enriched PUFAs in LTL muscle.

**Supplementary Information:**

The online version contains supplementary material available at 10.1007/s44154-024-00182-w.

## Introduction

The production performance of farm animals is significantly influenced by the climatic environment, and global warming has led to increasingly frequent heat waves and high ambient temperatures, which pose a severe constraint for livestock production. Additionally, genetic selection for production traits, including lean tissue accretion for pigs, makes farm animals more vulnerable to heat stress due to their higher metabolic heat production (Baumgard and Rhoads Jr 2013). Because of their limited capacity to dissipate heat and scattered sweat glands, pigs are particularly susceptible to high temperatures. Heat-stressed pigs exhibit reduced feed intake, negatively impacting growth and production performance, particularly carcass and meat quality (Baumgard and Rhoads Jr 2013). Heat stress in barrows produced contaminated carcasses characterized by more lean tissue and less back-fat thickness (Cruzen et al. 2015). *Longissimus thoracis et lumborum* (LTL) muscle from heat-stressed pigs tends to have higher extraordinary lightness (L^*^) value and lower redness (a^*^) value, higher drip loss, and high ambient temperature increased the risk for the prevalence of pale, soft, and exudative (PSE) meat (Yang et al. 2014). Changes in the chemical composition of LTL muscle, such as the decline in intramuscular fat (IMF) content, have also been documented in heat-stressed pigs (Ma et al. 2019). The main mechanisms underlying high temperature-induced meat quality deterioration are an imbalance between pro-oxidant and antioxidant capacity and muscle fiber transformation (Yang et al. 2014; Yin et al. 2022).

Time-restricted feeding (TRF) is a short-term fasting regime that allows subjects to consume within a specific time window during a day (Sutton et al. 2018). Typically, food consumed for less than 10 h per day is required for short-term TRF, representing a practical approach to control dietary intake by extending fasting time. Recently, an increasing number of studies have demonstrated the beneficial effects of TRF on metabolic disorders. TRF could prevent high-fat diet-induced obesity, fatty liver, and glucose intolerance in mice (Chaix et al. 2018), decrease body weight and improve insulin sensitivity and beta cell responsiveness in humans with pre-diabetes (Sutton, et al. 2018), and suppress the inflammatory response and oxidative stress in the colon tissues of the colitis mice (Zhang et al. 2020). Study on skeletal muscle function in obesity drosophila have also revealed that TRF improves muscle physiology and alleviates muscle dysfunction-related phenotypes (Villanueva et al. 2019). Furthermore, it is worth noting that TRF also makes positive impacts on farm animals, including improved daily weight gain and feed efficiency in fattening geese (Lui et al. 2014), increased carcass weight and reduced cooking loss of quails (Elbaz et al. 2022). Nevertheless, the impact of TRF on pig growth performance and meat quality of LTL muscle remains under investigation.

As one of the most critical regulator of intracellular redox balance, nuclear factor erythroid 2-related factor 2 (Nrf2) and its target gene heme oxygenase-1 (HO-1) were usually activated in response to oxidative stress (Itoh et al. 1997). After activation, cytoplasmic Nrf2 is transferred to the nucleus and binds to the antioxidant response element in the promoter of target genes to initiate the expression of multiple antioxidant factors, including HO-1, NAD (P)H/quinone oxidoreductase 1 (NQO1) and glutamate-cysteine ligase catalytic subunit (GCLC). Even previous studies found that high temperature induced heat stress in farm animals was accompanied bythe activation of Nrf2 signaling pathway, chronic heat stress suppressed this cascade (Zhao et al. 2022). The mechanism underlying the TRF displayed antioxidant capacity in a couple of inflammatory models has not been elucidated yet. Thus, we hypothesize that TRF relieves the high temperature induced oxidative damage and deleterious effect on meat quality in LTL muscle of growing pigs through an activation of Nrf2/HO-1 antioxidant pathway.

## Results

### Time-restricted feeding increased the body weight and feed intake, and heat treatment reduced the loin eye muscle area

During the trial, pigs in the TRF group showed the highest body weights (BWs) from day 27 (August 14th) to the end of the trial (Fig. S1A). The variation curve of BWs was generally in line with the feed intake that TRF group presented the highest daily feed intake from day 33 (August 20th) to day 51 (September 9th) (Fig. S1B). Interaction effects between treatments on average daily weight gain (ADWG, Table 1, *P* < 0.01) and BWs before slaughter (*P* = 0.05) were observed, with a significantly higher ADWG in TRF group compared to the thermal neutral group (NT) and heat treatment (HT) and TRF co-treatment group (HT + TRF, *P* < 0.05). HT reduced the corrected loin eye muscle area (*P* < 0.05), and pigs in HT + TRF group exhibited the least area among groups (Table 1).

**Table 1:** Effects of time-restricted feeding and heat exposure on bodyweight and carcass quality of growing pigs

Item	-TRF^1^		+ TRF		SED	Main effect, HT		Main effect, TRF		*P*-value		
	NT	HT	NT	HT		NT	HT	-TRF	+ TRF	HT	TRF	HT × TRF
Initial BWs (kg)	12.83	12.08	13.88	13.53	0.87	13.36	12.81	12.46	13.71	0.54	0.17	0.82
ADWG (kg)	0.43^b^	0.47^ab^	0.49^a^	0.43^b^	0.01	0.46	0.45	0.45	0.46	0.39	0.53	< 0.01
BWs before slaughter (kg)	39.23	40.02	43.45	38.62	1.56	41.34	39.29	39.63	41.01	0.15	0.31	0.05
Carcass length (cm)	77.33	80.42	83.33	78.60	2.18	80.33	79.51	78.88	80.97	0.71	0.35	0.09
Corrected LEMA (cm^2^)	53.98	49.9	51.80	44.74	2.26	52.89^a^	47.32^b^	51.94	48.27	0.02	0.12	0.52

Means within a row with different superscript letters are significantly different (*P* < 0.05). The results were presented as mean values with SED (standard error of difference, *n* = 6)

^1^*TRF* time restrict feeding, *NT* thermal neutral. *HT* heat treatment, *BWs* body weights, *ADWG* average daily weight gain, *LEMA* loin eye muscle area. Same for the following tables

### Time-restricted feeding restored the decline in meat quality of LTL muscle caused by high ambient temperature exposure

HT significantly increased the shear force and 24 h drip loss of LTL muscle (Table 2, *P* < 0.01), while treatment of TRF significantly declined them (*P* < 0.01). HT group exhibited higher shear force and drip loss than the others (*P* < 0.05), while TRF group presented the least shear force and drip loss (*P* < 0.05). No difference in cooking loss and water holding capacity (WHC) between groups (*P* > 0.05). HT tended to increase the lightness (L^*^) of LTL muscle (Table 2, *P* = 0.07), and there was significant interaction effect on L^*^ between treatments. Treatment of TRF faded the muscle redness (a^*^, *P* < 0.01). Significantly increased L^*^ in HT group and reduced a^*^ in TRF group were observed compared to those in NT group, respectively (*P* < 0.05).

**Table 2:** Effects of time-restricted feeding and heat exposure on meat quality and oxidative status of *longissimus thoracis et lumborum* muscle in growing pigs

Item^1^	-TRF		+ TRF		SED	Main effect, HT		Main effect, TRF		*P*-value		
	NT	HT	NT	HT		NT	HT	-TRF	+ TRF	HT	TRF	HT × TRF
Shear force (N)	27.22^b^	32.68^a^	22.48^c^	25.45^bc^	1.14	24.85^b^	29.06^a^	29.95^a^	23.97^b^	< 0.01	< 0.01	0.16
Cooking loss (%)	45.45	44.34	45.03	45.22	0.49	45.24	44.78	44.89	45.13	0.34	0.18	0.63
24 h Drip loss (%)	5.60^b^	7.24^a^	4.89^b^	5.23^b^	0.31	5.24^b^	6.24^a^	6.42^a^	5.06^b^	< 0.01	< 0.01	0.05
WHC (%)	36.10	37.47	38.15	38.45	1.07	37.12	37.96	36.78	38.30	0.36	0.11	0.55
Meat color												
Lightness L*	52.70^b^	55.32^a^	53.39^ab^	53.12^ab^	0.63	53.05	54.22	54.01	53.26	0.07	0.25	0.03
Redness a*	6.13^a^	6.18^a^	4.09^b^	4.75^ab^	0.49	5.11	5.46	6.16^a^	4.42^b^	0.48	< 0.01	0.54
Yellowness b*	6.22	6.18	6.37	5.96	0.57	6.30	6.07	6.20	6.17	0.66	0.95	0.72
Oxidative status												
MDA (nmol/mg prot)	0.24^b^	0.35^a^	0.27^ab^	0.24^b^	0.04	0.25	0.30	0.29	0.25	0.13	0.18	0.02
GSH-Px (U/mg prot)	4.58^b^	10.55^a^	4.08^b^	6.24^ab^	1.58	4.33^b^	8.40^a^	7.56	5.16	0.02	0.15	0.25
CAT (U/mg prot)	1.10	1.12	0.90	0.47	0.27	1.00	0.80	1.11	0.69	0.35	0.07	0.31
T-SOD (U/mg prot)	7.92	7.54	8.06	8.43	0.43	7.99	7.98	7.73	8.24	0.99	0.24	0.38

Means within a row with different superscript letters are significantly different (*P* < 0.05). The results were presented as mean values with SED (standard error of difference, *n* = 6)

^1^*MDA* malondialdehyde, *GSH-Px* glutathione peroxidase, *CAT* catalase, *T-SOD* total superoxide dismutase

### Time-restricted feeding improved the antioxidant capacity and expressions of Nrf2 anti-oxidative cascade in LTL muscle that were reduced by heat exposure

HT significantly increased the activity of glutathione peroxidase (GSH-Px) and an interaction effect on malondialdehyde (MDA) content in LTL muscle between treatments was found (Table 2, *P* < 0.05). There was an accumulation of MDA in HT group compared to NT and HT + TRF group (*P* < 0.05), and GSH-Px activity was also higher in HT group compared to NT group (*P* < 0.05). No difference in the activities of catalase (CAT) and total superoxide dismutase (T-SOD) between the groups (*P* > 0.05).

**Fig. 1:**
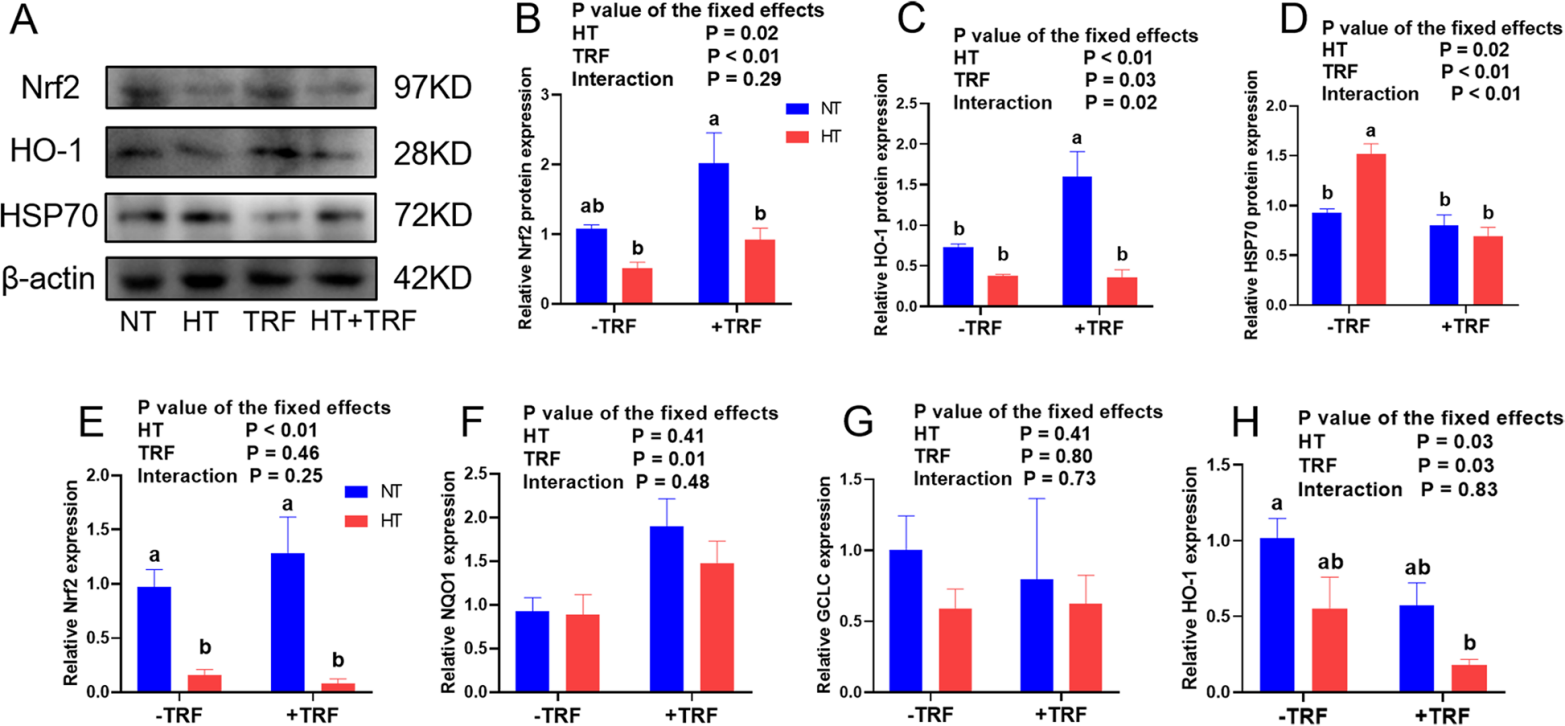
Effects of heat treatment (HT) and time-restricted feeding (TRF) on the expression of Nrf2/HO-1 antioxidant pathway in *Longissimus thoracis et lumborum* muscle. **A-D** Western blot analysis of Nrf2, HO-1 and HSP70 protein levels normalized to β-actin and quantification of relative protein densitometry. **E–H** Relative mRNA expressions of *Nrf2*, *NQO1*, *GCLC* and *HO-1* normalized to *GAPDH* expression. Data were presented as mean ± SEM. *P* value for the effects of HT, TRF and HT × TRF interaction. ^a.b.c^ Columns with different superscript letters are significantly different (*P* < 0.05)

Nrf2 and its target genes, mainly antioxidants factors including HO-1, NQO1 and GCLC, were investigated in LTL muscle. The results showed a significantly suppressed Nrf2 and HO-1 expressions by the chronic HT in both protein and mRNA levels (Fig. 1A, B, C, E, H, *P* < 0.05), while treatment of TRF enhanced the protein expressions of the pathway (Fig. 1A, B, C, *P* < 0.05). Protein expressions of HSP70 was improved by HT, and this phenotype was alleviated by treatment of TRF, with a significant interaction effect between treatments (Fig. 1D, *P* < 0.05). TRF increased the mRNA expression of *NQO1* (Fig. 1F, *P* < 0.05), and no difference was observed between treatments in *GCLC* gene expression (Fig. 1G). TRF group displayed the highest protein levels of Nrf2 and HO-1 between groups, and HSP70 expression was higher in HT group than others (*P* < 0.05).

### Time-restricted feeding altered the chemical composition of LTL muscle, and heat treatment increased the intramuscular hydroxyproline content

Treatment of TRF resulted in a significant increase in moisture content (*P* < 0.05), and a decrease in glycogen contents in LTL muscle (Table 3, *P* < 0.01). HT significantly declined the glycogen content and increased the hydroxyproline content (*P* < 0.05), and significant interaction effect on hydroxyproline content was revealed between treatments (*P* < 0.05). The content of muscle glycogen in all three treatment groups was lower than NT group (*P* < 0.05). HT group displayed higher hydroxyproline concentration than that in NT group (*P* < 0.05), while it was diminished in HT + TRF group compared to the HT group (*P* < 0.05). Both treatments made no difference in lactic acid content and metabolic enzyme activities including succinic dehydrogenase (SDH), malate dehydrogenase (MDH), and lactate dehydrogenase (LDH) in LTL muscle (Table S3).

**Table 3:** Effects of time-restricted feeding and heat exposure on bodyweight, carcass quality and *longissimus thoracis et lumborum* muscle chemical composition of growing pigs

Item	-TRF		+ TRF		SED	Main effect, HT		Main effect, TRF		*P*-value		
	NT	HT	NT	HT		NT	HT	-TRF	+ TRF	HT	TRF	HT × TRF
Moisture (%)	74.42	74.76	75.60	75.42	0.45	75.01	75.09	74.59^b^	75.51^a^	0.85	0.04	0.54
Total protein (%)	21.29	21.15	21.41	21.46	0.48	21.35	21.31	21.22	21.43	0.93	0.65	0.84
Glycogen (mg/g)	1.12^a^	0.89^b^	0.86^b^	0.78^b^	0.05	0.99^a^	0.84^b^	1.01^a^	0.82^b^	0.01	< 0.01	0.15
Hydroxyproline (mg/g)	0.40^b^	0.64^a^	0.47^ab^	0.45^b^	0.05	0.44^b^	0.54^a^	0.52	0.46	0.04	0.21	0.01
Lactate (mmol/gprot)	1.16	1.17	1.14	1.26	0.14	1.15	1.21	1.16	1.20	0.67	0.82	0.68

Means within a row with different superscript letters are significantly different (*P* < 0.05). The results were presented as mean values with SED (standard error of difference, *n* = 6)

### Time-restricted feeding reversed the transformation of muscle *fiber* and decline in expression of muscle growth-related genes induced by heat exposure

**Fig. 2:**
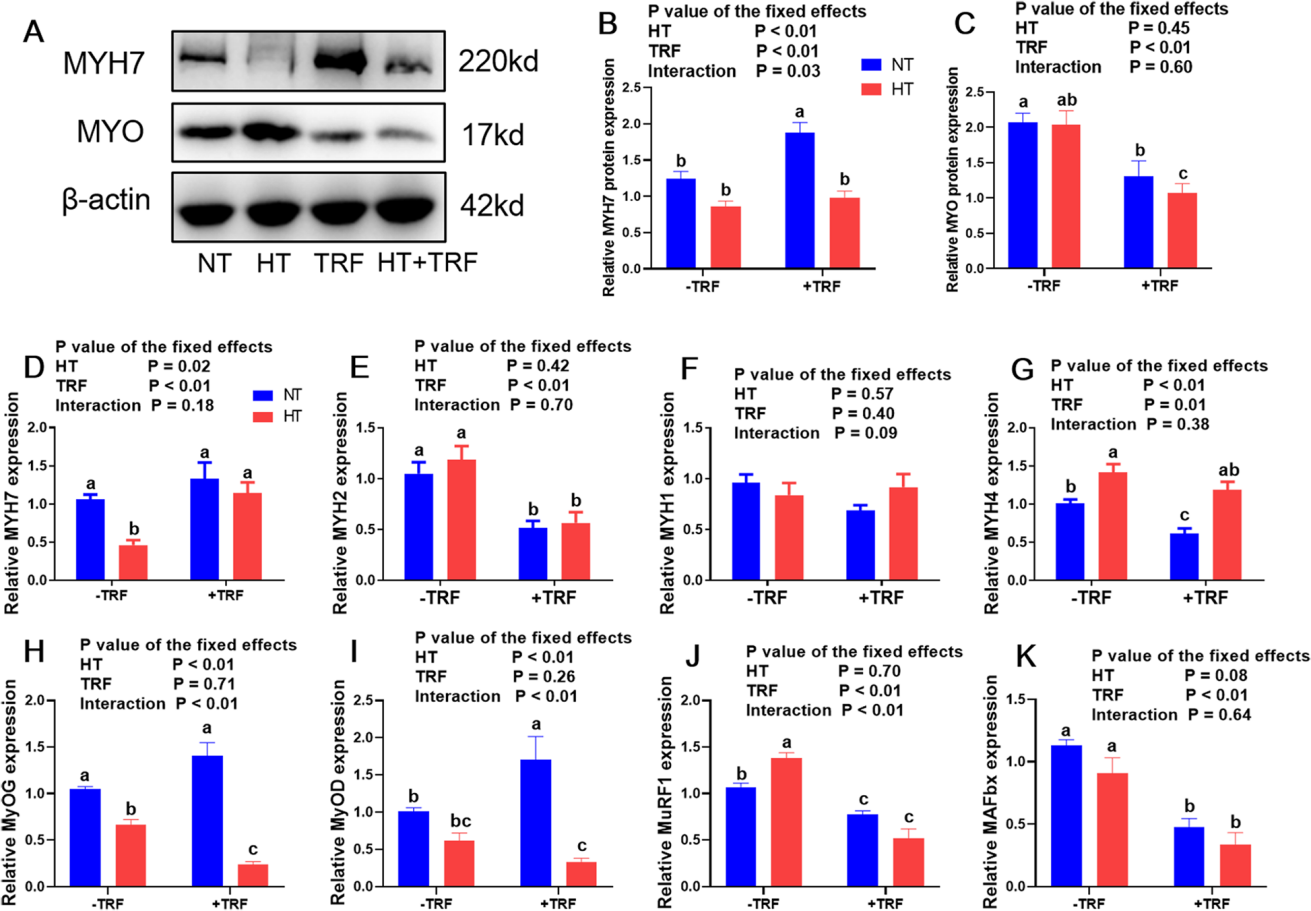
Effects of heat treatment (HT) and time-restricted feeding (TRF) on muscle fiber type-related and growth-related gene expressions in *Longissimus thoracis et lumborum* muscle. **A-C** Western blot analysis of MYH7 and MYO protein levels normalized to β-actin and quantification of relative protein densitometry. **D-K** Relative mRNA expressions of *MYH7*, *MYH2*, *MYH1*, *MYH4*, *MyOG*, *MyOD*, *MuRF1* and *MAFbx* normalized to *GAPDH* expression. Data were presented as mean ± SEM. *P* value for the effects of HT, TRF and HT × TRF interaction. ^a.b.c^ Columns with different superscript letters are significantly different (*P* < 0.05)

As the transcripts are closely related to the amount of the corresponding muscle fibers, qRT-PCR was performed to determine muscle fiber composition (Lefaucheur et al. 2002). HT significantly decreased the transcript levels of myosin heavy chain7 (*MYH7*, Fig. 2D, *P* < 0.05) and increased the expression of *MYH4* (Fig. 2G, *P* < 0.01), while treatment of TRF reversed these variation (*P* < 0.05). The HT group presented lower expression of *MYH7* gene than the others (*P* < 0.05) and up-regulated expression of *MYH4* gene compared to the NT group (*P* < 0.05). The *MYH4* gene expression in TRF group was the lowest among groups (*P* < 0.05). Moreover, treatment of TRF significantly declined the expression of *MYH2* (Fig. 2E, *P* < 0.01), with lower expression in two TRF groups than these in non-TRF groups (*P* < 0.05). No effect of both treatments on *MYH1* gene expression was observed (Fig. 2F, *P* > 0.05). MYH7 protein expression was in parallel with the mRNA transcript level, being significant declined by HT and increased by treatment of TRF (Fig. 2A, B, *P* < 0.01). There was also a significant interaction effect on MYH7 protein level between treatments (*P* < 0.05), with the highest protein level in TRF group than others (*P* < 0.05). Treatment of TRF significantly decreased the myoglobin protein level (Fig. 2A, C, *P* < 0.01), with significant decline in two TRF groups compared to the NT group (*P* < 0.05).

HT decreased the transcript levels of muscle growth-related genes myogenic (*MyOG*) and myogenic differentiation (*MyOD*, Fig. 2H, I, *P* < 0.01), coupled with interaction effect between treatments (*P* < 0.01). The *MyOG* mRNA expression was down-regulated in two HT groups compared to the NT group (*P* < 0.05). The TRF group displayed improved *MyOD* expression compared to the NT group (*P* < 0.05), while it was declined in HT + TRF group (*P* < 0.05). Treatment of TRF significantly diminished the expressions of muscle-specific RING-finger protein 1 (*MuRF1*) and atrophy-related genes atrogen-1 (*MAFbx*) gene, and interaction effect on *MuRF1* expression was revealed between treatments (Fig. 2J, K, *P* < 0.01). The HT group showed the highest mRNA expression of *MuRF1* among groups (*P* < 0.05). The *MuRF1* and *MAFbx* expressions in both TRF groups were significantly reduced compared to these in NT group (*P* < 0.05).

### Time-restricted feeding enriched the polyunsaturated fatty acids in LTL muscle that was reduced by high ambient temperature exposure

Saturated fatty acids (SFAs), mono- and polyunsaturated fatty acids (MUFAs, PUFAs) were measured to determine the effects of HT and TRF on the fatty acids composition of LTL muscle (Table 4). Pigs treated with TRF showed decreased intramuscular fat content in LTL muscle (*P* < 0.05) that HT + TRF group displayed the lowest one among groups. HT decreased the proportions of heptadecamonoenoic acid (c17:1, *P* < 0.05) and trans-oleic acid (c18:1n9t, *P* < 0.05), with significant interaction effects between treatments on these acids (*P* < 0.01, *P* < 0.05). TRF group showed the highest proportions of c17:1 and c18:1n9t among groups, while they were significantly reduced in HT + TRF group compared to the TRF group (*P* < 0.05). Treatment of TRF slightly declined the proportions of arachidic acid (c20:0, *P* < 0.05) and eicosaenoic acid (c20:2, *P* < 0.05), and interaction effects on the content of these acids were observed between treatments (*P* < 0.01, *P* = 0.05). Proportions of c20:0 and c20:2 were lower in TRF group compared to the NT group (*P* < 0.05). There was significant interaction effect on eicosatrienoic acid (c20:3n6, *P* < 0.05) and arachidonic acid (c20:4n6, *P* < 0.05) proportions between treatments that both HT group and TRF group (*P* < 0.05) displayed increased proportions of these acids, while in HT + TRF group the enrichments were reduced. Treatment of TRF increased the proportion of total SFAs (*P* < 0.05) and reversely declined the sum of MUFAs (*P* < 0.05), coupled with significant interaction effects on these two sums between treatments (*P* < 0.05). HT + TRF group showed the most enriched SFAs among groups (*P* < 0.05), and TRF group displayed a lower sum of MUFAs than that in NT group (*P* < 0.05). The sum of PUFAs and n6 fatty acids were significantly reduced by HT (*P* < 0.05), coupled with interaction effects between treatments (*P* < 0.01). Proportions of PUFAs and n6 fatty acids were higher in TRF group compared to the NT and HT + TRF groups (*P* < 0.05). These fluctuation resulted in reduced ratio of PUFAs/SFAs (*P* < 0.05) by treatment of HT. Interaction effect on ratio of PUFAs/SFAs was identified between treatments (*P* < 0.01) that TRF group displayed the highest ratio among groups (*P* < 0.05).

**Table 4:** Intramuscular fat (IMF) content and (fatty acids) FAs composition (% of total FA) of the *longissimus thoracis et lumborum* muscle in growing pigs

Item^1^	-TRF		+ TRF		SED	Main effect, HT		Main effect, TRF		*P*-value		
	NT	HT	NT	HT		NT	HT	-TRF	+ TRF	HT	TRF	HT × TRF
IMF, mg/100 g tissue	3747^a^	3428^ab^	2944^ab^	2735^b^	247	3345	3082	3588^a^	2839^b^	0.25	< 0.01	0.80
c14:0	0.88	0.88	0.83	0.92	0.02	0.85	0.90	0.88	0.87	0.24	0.87	0.29
c16:0	22.00	21.96	22.30	22.71	0.39	22.15	22.33	21.98	22.51	0.65	0.19	0.57
c16:1 cis-9	2.03	2.21	2.15	2.15	0.22	2.09	2.18	2.12	2.15	0.67	0.90	0.66
c17:0	0.25	0.23	0.28	0.24	0.02	0.26	0.24	0.24	0.26	0.13	0.19	0.51
c17:1 cis-10	0.69^b^	0.82^ab^	1.09^a^	0.24^c^	0.12	0.89^a^	0.53^b^	0.75	0.66	< 0.01	0.37	< 0.01
c18:0	12.82	12.28	12.28	12.95	0.52	12.55	12.62	12.55	12.62	0.89	0.89	0.22
c18:1 trans-9	0.10^ab^	0.10^ab^	0.12^a^	0.08^b^	0.01	0.11^a^	0.09^b^	0.10	0.10	0.03	0.72	0.03
c18:1 cis-9	32.22	32.20	30.27	32.49	1.29	31.25	32.35	32.21	31.38	0.34	0.47	0.33
c18:2 cis-9,cis-12	16.54	17.24	16.66	15.58	0.75	16.60	16.41	16.89	16.12	0.72	0.16	0.11
c18:3n6	0.09	0.11	0.13	0.10	0.01	0.11	0.11	0.10	0.12	0.90	0.35	0.11
c20:0	0.18^a^	0.15^b^	0.14^b^	0.16^ab^	0.01	0.16	0.16	0.17^a^	0.15^b^	0.66	0.04	< 0.01
c20:2	0.61^a^	0.57^ab^	0.49^b^	0.56^ab^	0.03	0.55	0.56	0.59^a^	0.53^b^	0.67	0.04	0.05
c20:3n6	0.38^a^	0.47^ab^	0.53^b^	0.43^ab^	0.04	0.45	0.45	0.42	0.48	0.86	0.19	0.02
c20:4n6	2.50^b^	3.09^ab^	3.71^a^	2.40^b^	0.32	3.11	2.75	2.80	3.06	0.21	0.36	< 0.01
c24:0	0.11	0.14	0.15	0.12	0.02	0.13	0.13	0.12	0.14	0.96	0.48	0.16
c22:6	0.26	0.28	0.30	0.25	0.03	0.28	0.26	0.27	0.28	0.57	0.80	0.20
∑SFAs^1^	36.24^ab^	35.77^b^	35.98^b^	38.02^a^	0.49	36.11	36.90	36.01^b^	37.00^a^	0.10	0.04	0.01
∑MUFAs^1^	36.6^a^	35.77^ab^	31.74^b^	35.57^ab^	1.07	34.17	35.67	36.19^a^	33.65^b^	0.18	0.03	0.04
∑PUFAs^1^	20.6^b^	21.52^ab^	24.15^a^	19.58^b^	0.78	22.37^a^	20.55^b^	21.06	21.86	0.03	0.32	< 0.01
∑n-6 PUFAs	19.13^bc^	21.01^ab^	22.81^a^	18.24^c^	0.49	20.97^a^	19.63^b^	20.07	20.52	0.02	0.37	< 0.01
∑MUFAs/∑SFAs	1.00	1.00	0.88	0.96	0.04	0.94	0.98	1.00	0.92	0.07	0.28	0.34
∑PUFAs/∑SFAs	0.56^b^	0.59^b^	0.68^a^	0.52^b^	0.02	0.62	0.55	0.58^b^	0.60^a^	0.31	< 0.01	< 0.01

Means within a row with different superscript letters are significantly different (*P* < 0.05). The results were presented as mean values with SED (standard error of difference, *n* = 6)

^1^∑SFAs, saturated fatty acids and is a sum of c14:0, c16:0, c17:0, c18:0, c20:0 and c24:0. ∑MUFAs, monounsaturated fatty acid and is a sum of c16:1, c17:1, c18:1n9t and c18:1n9c. ∑PUFAs, polyunsaturated fatty acids and is a sum of c18:2n6c, c18:3n6, c20:2, c20:3n6, c20:4n6 and c22:6

**Fig. 3:**
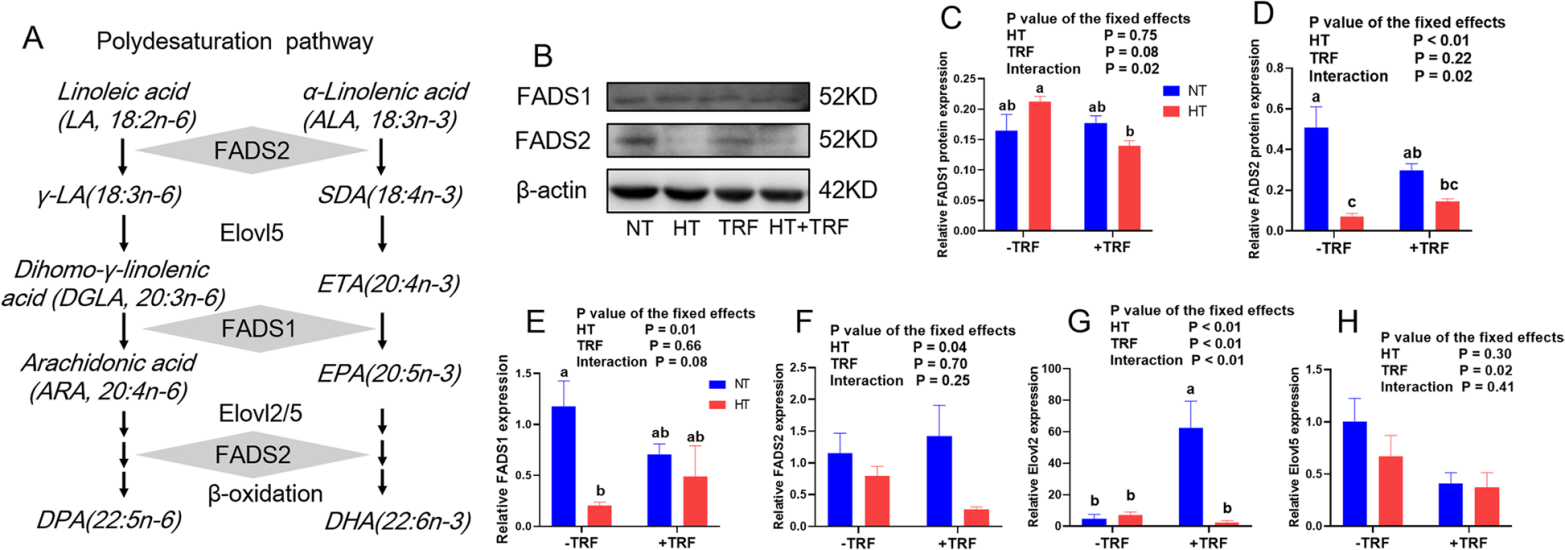
Effects of heat treatment (HT) and time-restricted feeding (TRF) on the expression of genes involved in polydesaturation in *Longissimus thoracis et lumborum* muscle. **A** Schematic overview of fatty acid metabolic pathways catalyzing synthesis of PUFAs. **B-D** Western blot analysis of FADS1 and FADS2 protein levels normalized to β-actin and quantification of relative protein densitometry. **E–H** Relative mRNA expressions of *FADS1*, *FADS2*, *Elovl2* and *Elovl5* normalized to *GAPDH* expression. Data were presented as mean ± SEM. *P* value for the effects of HT, TRF and HT × TRF interaction. ^a.b.c^ Columns with different superscript letters are significantly different (*P* < 0.05)

In general, most PUFAs in mammals are considered to be derived from exogenous sources as mammals lack an enzyme for the de novo synthesis. Linoleic acid (LA, 18:2n-6) and a-linolenic acid (ALA, 18:3n-3) are essential fatty acids that are initially subjected to several desaturation events mediated by fatty acid desaturase 1 (FADS1) and FADS2, and then elongation of acyl tails by elongation-of-very-long-chain-fatty acids 2 (Elovl2/5) to generate other highly unsaturated fatty acids (Fig. 3A). Here we observed a significant decline in FADS2 protein expression caused by HT, and interaction effect between treatments was revealed in protein expressions of FADS1 and FADS2 (Fig. 3B, D, *P* < 0.05). As regard to gene expressions, HT significantly reduced mRNA expressions of *FADS1*, *FADS2* and *Elovl2* (Fig. 3E, F, G, *P* < 0.05). Treatment of TRF improved the mRNA levels of *Elovl2* and reduced the transcript levels of *Elovl5* (Fig. 3G, H, *P* < 0.05). HT group displayed the lowest protein expression of FADS2 and gene expression of *FADS1* between groups. Gene expression of *Elovl2* in TRF group was the highest among groups.

### Time-restricted feeding and heat treatment altered the amino acids composition of LTL muscle

Essential amino acids (EAA) and NEAA contents of LTL muscle were evaluated between treatments (Table 5). HT significantly increased the contents of methionine (Met, *P* < 0.05), asparagine (Asp, *P* < 0.05) and cysteine (Cys, *P* < 0.01). The HT group presented the highest contents of Met (*P* < 0.05) and Asp among groups, and Cys content in HT + TRF group was higher than that in TRF group (*P* < 0.05). Proline (Pro) content was reduced by treatment of TRF (*P* < 0.05) coupled with an interaction effect between treatments (*P* < 0.05). The TRF group displayed a lower Pro content than that in NT group (*P* < 0.05). No additional difference in the amino acid profile between groups (*P* > 0.05).

**Table 5:** The amino acid composition of longissimus thoracis et lumborum muscle samples in growing pigs (g/100 g fresh meat)

Item	-TRF		+ TRF		SED	Main effect, HT		Main effect, TRF		*P*-value		
	NT	HT	NT	HT		NT	HT	-TRF	+ TRF	HT	TRF	HT × TRF
EAA^1^												
Threonine (Thr)	1.00	1.07	1.07	1.05	0.04	1.04	1.06	1.03	1.06	0.51	0.38	0.17
Valine (Val)	1.04	1.06	1.03	1.09	0.03	1.03	1.08	1.05	1.06	0.14	0.62	0.40
Methionine (Met)	0.57^b^	0.64^a^	0.58^ab^	0.62^ab^	0.02	0.58^b^	0.63^a^	0.60	0.60	< 0.01	0.77	0.37
Isoleucine (Ile)	0.98	1.00	0.97	1.02	0.03	0.98	1.01	0.99	0.99	0.22	0.88	0.50
Leucine (Leu)	1.74	1.83	1.79	1.83	0.06	1.76	1.83	1.79	1.81	0.19	0.70	0.59
Phenylalanine (Phe)	0.83	0.86	0.85	0.86	0.03	0.84	0.86	0.84	0.86	0.57	0.35	0.50
Lysine (Lys)	1.91	2.02	1.95	2.01	0.07	1.93	2.01	1.97	1.98	0.15	0.79	0.64
Histidine (His)	0.89	0.88	0.88	0.89	0.03	0.88	0.88	0.89	0.88	0.95	0.88	0.76
Argnine (Arg)	1.37	1.43	1.38	1.42	0.04	1.38	1.42	1.40	1.40	0.19	0.91	0.88
Total EAA	10.34	10.76	10.51	10.78	0.35	10.43	10.77	10.55	10.65	0.22	0.73	0.78
NEAA^1^												
Asparagine (Asp)	2.03^ab^	2.15^a^	1.96^b^	2.10^ab^	0.06	1.99	2.13	2.09	2.03	0.01	0.25	0.86
Serine (Ser)	0.81	0.89	0.89	0.85	0.02	0.85	0.87	0.85	0.87	0.50	0.33	0.01
Glutamic acid (Glu)	3.43	3.64	3.56	3.62	0.12	3.50	3.63	3.54	3.59	0.16	0.58	0.42
Glycine (Gly)	1.08	1.01	0.96	1.02	0.04	1.02	1.02	1.05	0.99	0.98	0.15	0.10
Alanine (Ala)	1.23	1.26	1.24	1.29	0.04	1.24	1.27	1.25	1.26	0.21	0.64	0.69
Cysteine (Cys)	0.08^ab^	0.08^ab^	0.04^b^	0.11^a^	0.01	0.06^b^	0.10^a^	0.08	0.07	< 0.01	0.57	0.02
Tyrosine (Tyr)	0.63	0.66	0.63	0.66	0.02	0.63	0.66	0.64	0.65	0.20	0.98	0.87
Proline (Pro)	0.58^a^	0.49^ab^	0.43^b^	0.48^ab^	0.03	0.50	0.49	0.54^a^	0.45^b^	0.64	0.01	0.02
EAA/NEAA	1.02	1.04	1.05	1.03	0.01	1.04	1.04	1.03	1.04	0.81	0.42	0.11

Means within a row with different superscript letters are significantly different (*P* < 0.05). The results were presented as mean values with SED (standard error of difference, *n* = 6)

^1^*EAA* essential animo acids. Total EAA was a sum of the concentrations of all the essential amino acids in the present table. *NEAA* non essential animo acids. Total NEAA was a sum of the concentrations of all the non essential amino acids in the present table

## Discussion

Prolonged exposure to high ambient temperature in pigs has been proved to be detrimental to growth performance and meat quality. Prior study has reported that PSE pork is more frequent in summer than the other seasons, characterized by pale color and increased drip loss (Cobanovic et al. 2020). Time-restricted feeding requires food consumption within a limited time window during the day. Studies in animals and humans have manifested various beneficial effects of TRF (Lundell et al. 2020; Zhang et al. 2020), such as improved production performance and meat quality in geese and quail (Elbaz et al. 2022; Lui et al. 2014). Given the fact that growing pigs are more vulnerable to high temperature and deficiency of reports on the influence of TRF on growth performance and muscle development of pigs, growing pigs were employed in the current study. The results showed that pigs treated with TRF displayed higher daily weight gain and improved meat quality of the *Longissimus thoracis et lumborum* muscle, mitigating the detrimental effects of high-temperature exposure. These beneficial effects of TRF were associated with restored Nrf2/HO-1 anti-oxidative capacity, modified muscle development and fiber composition, and increased proportion of PUFAs in the LTL muscle. Further studies are needed to fully validate the TRF-induced improvement on meat quality of LTL muscle in fattening pigs raised in high ambient temperature.

It is widely accepted that high ambient temperature reduces pigs' feed intake and daily gain. Even though consistent heat exposure at 30 °C in growing pigs reduced the feed intake by 20% (Pardo et al. 2021), in our study a 4 h 35 °C heat exposure failed to alter the feed consumption and weight gain, which may due to the fact that pigs consumed more feed during the thermal neutral period. Mammals treated with TRF learn to consume comparable amounts of food in the restricted time interval to that consumed with ad libitum feeding within days (Currenti et al. 2021). This is in agreement with our study that feed intake in two TRF groups was comparable to non-TRF groups in the first 30 days of the trial. Interestingly, pigs in TRF group achieved more consumption in the last 24 days, resulted in the highest ADWG and BWs among groups. The improvement effects of TRF were abolished by HT as the HT + TRF group showed the lowest final BWs. Loin muscle area is a well-known biomarker for carcass quality of pigs such as carcass lean rate and backfat thickness, and is influenced by variations in the nutrient and growth environment (Yang et al. 2015). Our study confirmed that HT reduced loin muscle area of LTL muscle.

Continuous heat exposure at 30 °C in pigs for three weeks increased the shear force of LTL muscle (Yang et al. 2014), being consistent with our result that HT group displayed the highest shear force among groups. Variation in shear force is partially due to the collagen content in muscle since collagen is a fibrous structural protein, major constituent of muscle connective tissue and responsible for muscle strengthening (Bosselmann et al. 1995). 4-Hydroxyproline is the main component of collagen, being traditionally employed as an indicator of muscular collagen content (Liscio and Hopley 2016). Here we observed that HT increased the hydroxyproline content in LTL muscle, which might contribute to the increased shear force. Furthermore, HT also weakened the WHC as indicated by hastened 24 h drip loss, resulting in an exudative meat with poor palatability. TRF reduced the shear force and 24 h drip loss, and increased the moisture of LTL muscle, implying that TRF mitigated the detrimental effects on meat quality caused by high ambient temperature. After animals are harvested, cessation of blood flow and oxygen supply makes intramuscular glycogen as the main ATP source through anaerobic glycolysis. Pigs continuously exposed to 35 °C showed reduced feed intake and glycogen content in LTL muscle (Ma et al. 2019). Even though there was no reduction of feed intake in our results, both treatments of HT and TRF declined the glycogen content in LTL muscle.

Meat color is an important parameter for rating sensory quality. A bright red color always complies with consumer preference, being attributed to the light-scattering matrix of cellular material, myofibrillar proteins, connective tissue, and light-absorbing pigments (especially myoglobin) (MacDougall 1970). Pigs suffered from HT showed increased risks of PSE meat, characterized by increased L^*^ value (Yang et al. 2014). Our results revealed that HT increased the L^*^ value of LTL muscle at 24 h postmortem, being consistent with studies in autochthonous and commercial pigs (Cui et al. 2018; Simonetti et al. 2018). Myoglobin is an oxygen-binding protein in muscle that gives the characteristic red color of meat, and myoglobin content has been proved to be positively correlated with pork redness (a^*^) (Kim et al. 2010). This phenotype was confirmed in the current study that pigs treated with TRF possessed a lower myoglobin content and a^*^ value of LTL muscle.

Meat quality is affected by muscle fiber composition, which is categorized into various types including slow-twitch oxidative fiber (type 1, red fiber), fast-twitch oxidative fiber (type 2a, red fiber), fast-twitch glycolytic fiber (type 2b, white fiber), and fast-twitch oxidative glycolytic fiber (type 2x, white fiber) (Xing et al. 2019). The myosins of muscle fibers are composed of varying myosin heavy chains and light chains, and the heavy myosins of LTL muscle have four distinct isoforms (1, 2a, 2 × and 2d), encoded by the family genes (MYH7, MYH2, MYH1 and MYH4). In newborn piglets, all muscle fibers are initially red type (1, 2a) and then transformed to white type (2x, 2b) in a regular sequence: 1 ↔ 2a ↔ 2x ↔ 2b (Pette and Staron 2001). Our study observed lower proportion of type 1 fiber and higher proportion of type 2b fiber in LTL muscle of pigs treated with HT, indicating an enhanced fiber-type transformation by high temperature. Given the variation in ATPase and contraction activities of muscle fibers, the composition of fibers is closely related to the postmortem metabolism rate, during which muscle is converted into meat, and thereby plays a vital role in meat quality. The proportion of type 1 fiber is negatively correlated with lightness and positively correlated with WHC in pigs, and higher proportion of type 2b fiber is associated with tougher, paleness, and lower WHC in LTL muscle (Ryu et al. 2008). The current study found that meat quality attributes in HT group, including increased shear force, drip loss and meat lightness, were closely related to the lower proportion of type 1 fiber and higher proportion of type 2b fiber. Moreover, fiber type is also related to IMF, such as the positive correlation between the percentage of red fiber and IMF, and the negative correlation between white fiber and IMF (Hwang et al. 2010). Here we confirmed this relationship that pigs treated with TRF displayed reduced content of IMF and proportion of type 2a fiber (red fiber) in LTL muscle. Additionally, TRF was recently identified to enhance adipocyte thermogenesis and mitigate fat deposition, which may clarify the diminished IMF in two TRF groups (Hepler et al. 2022).

Chronic heat stress always resulted in a suppressed antioxidant capacity and accumulation of free radicals. Meat is susceptible to oxidative deterioration due to high concentrations of unsaturated lipids, heme pigments, metal catalysts and a range of oxidizing agents (Falowo et al. 2014). Oxidative deterioration in meat manifests in various forms, including discoloration, development of off flavor, poor shelf life, nutrient and drip losses (Contini et al. 2014; Herrera et al. 2021). Heat exposure expedited the accumulation of MDA in LTL muscle of lean pigs due to decreased activity of antioxidant enzymes and increased production of free radicals (Yang et al. 2014). In broilers, long-term heat stress blocked the Nrf2 antioxidant pathways with diminished protein expression of Nrf2 and HO-1 in the liver and gene expressions of Nrf2 signaling pathway in skeletal muscle (Ding et al. 2023; Zhao et al. 2022). Our study confirmed these phenotypes by an increased muscle MDA content and reduced expressions of Nrf2 and HO-1 in the HT group. Recently, in various disease models TRF displayed antioxidant attributes. TRF in ionizing radiation-exposed mice eliminated the accumulation of ROS in bone marrow cells via increased cellular Nrf2 expressions and restored activities of GSH-PX, SOD and CAT in both serum and spleen (Huo et al. 2024). Furthermore, TRF also ameliorated oxidative stress by increasing Nrf2 transcriptional factor in the rat hippocampus in a pilocarpine-induced acute seizure model (Mercado-Gómez et al. 2023). The current study confirmed these phenotypes that TRF up-regulated the Nrf2/HO-1 antioxidant cascade and diminished the accumulation of MDA in the HT + TRF group.

Heat exposure causes excessive accumulation of reactive oxygen species and increases the risk of muscle atrophy, characterized by skeletal muscle breakdown and mitochondrial malfunction via increased expression of MAFbx and MuRF1 (Enoki et al. 2016). MyOD and MyOG involve in muscle cell proliferation and differentiation, of which the expressions were blocked in disease-induced muscle atrophy (Zhang et al. 2010). Dietary leucine supplementation in heat-stressed pigs reduced the oxidative stress and gene expression of MuRF1 coupled with increased MyOD and MyOG expressions in LTL muscle (Yin et al. 2022). Our research found decreased mRNA level of MyOD and MyOG, and increased MuRF1 expression in the HT group, suggesting a suppressed muscle development and increased likelihood of muscle atrophy. These damages may also be attributed to the diminished expression of Nrf2, as recent study in Nrf2 deficiency mice observed skeletal muscle atrophy and loss of muscle mass (Ahn et al. 2018). The TRF group displayed increased MyOG and MyOD expressions and decline in MAFbx and MuRF1 expressions, contributing to the well-developed muscle fibers. These improvements of TRF may rely on the enhanced Nrf2/HO-1 expressions and alleviated oxidative stress in LTL muscle (Zhang et al. 2020).

The intramuscular fat abundance and fatty acids saturation have crucial repercussions on meat quality, and a link between specific FA profiles and pork flavor has been established previously (Tikk et al. 2007). Furthermore, the ratio of PUFAs to SFAs is one the most reliable markers of the nutritional value of meat (Wood and Enser 1997). Long-term constant heat exposure usually reduced feed intake and IMF content in LTL muscle (Cui et al. 2018; Ma et al. 2019), while in natural conditions of diurnal temperature fluctuation (27.9 ± 3 °C), no influence on IMF content and PUFAs/SFAs ratio was observed in Large White pig (Rinaldo and Mourot 2001), being consistent with our results. In autochthonous breed of Iberian pig, continuously HT had no effects on FA composition in LTL muscle (Simonetti et al. 2018). The discrepancies in IMF content and ratio of PUFAs rely on the severity (both extent and acute vs. chronic) of the heat load and pig breed. The PUFAs are substances that initiate lipid acids oxidation, and increased content of these acids may enhance the antioxidant activity (Rant et al. 2019). This view was attested by prior study that chronic heat exposure in broilers induced accumulation of MDA, and decline in SOD activity and abundance of various PUFA in breast (Qu and Ajuwon 2018). The current study reveals higher contents of PUFAs in the TRF group, mainly the omega-6 fatty acids including c20:3n6 and c20:4n6, which may contribute to the improved antioxidant capacity and meat quality. Our results also demonstrate that protein expression of FADS2 and gene expression of FADS1, enzymes involve in fatty acid desaturation, are suppressed by HT in LTL muscle, while TRF partially rescues the expressions. These phenotypes were probably attributed to the oxidative stress as high-fat diet induced hepatic oxidative stress concomitantly with loss of enzyme desaturation activity (Valenzuela et al. 2015). Furthermore, the hepatic oxidative stress and decline in enzyme desaturation activity caused by high-fat diet were normalized by supplementation with the antioxidant hydroxytyrosol or with antioxidant-rich extra virgin olive oil (Rincón-Cervera et al. 2016; Valenzuela et al. 2017). Here TRF partially restores the expressions of FADS2 and FADS1, which may be related to the antioxidant attributes that TRF displayed in LTL muscle. Meanwhile, TRF also boosts the expression of Elovl2, which preferentially elongates C20-C22 PUFA. The promotion of poly-desaturation pathway and enrichment of PUFAs contribute to the TRF-induced improvement of meat quality in LTL muscle.

Amino acids also contribute to the specific flavor of meat and serve as a crucial indicator of meat's nutritional value and quality (Ma et al. 2020). Each amino acids have its flavor, among which methionine is bitter (Lee et al. 2016). Methionine restriction in rats has been proven to alleviate the oxidative stress and inflammatory response (Sanz et al. 2006). Weaning pigs fed with a methionine-restricted diet reduced serum methionine concentration and promoted the formation of type 1 fiber in LTL muscle (Wu et al. 2019). Our study observed mounting methionine content in LTL muscle of pigs treated with HT, which may contribute to the oxidative stress and reduced proportion of type 1 fiber. Proline and hydroxyproline contribute to the majority of total amino acids in collagen, and hydroxyproline is derived from the post-translational hydroxylation of proline in proteins (primarily collagen) (Liscio and Hopley 2016). Here we observed reduced content of proline in LTL muscle by TRF, which may contribute to the reduced shear force in TRF groups.

## Conclusions

In conclusion, our study is the first to provide evidence that implementing a time-restricted feeding regimen for growing pigs will alleviate the detrimental effects of high ambient temperatures on meat quality. The improvement effects include mitigating oxidative stress, modulating muscle fiber composition, and enriching the polyunsaturated fatty acids in LTL muscle. TRF restored the expression of Nrf2/HO-1 antioxidant pathway that was blocked by chronic HT in LTL muscle, and promoted the expression of vital genes involved in poly-desaturation pathway. These findings suggest that TRF is a potential practicable feeding regimen to alleviate the deleterious effects on meat quality caused by high ambient temperature.

## Materials and methods

### Animals and experimental design

A total of twenty-four Duroc × Landrace × Yorkshire (DLY) pigs (13 gilts and 11 castrated males, 11.0 ± 1.9 kg BW, at 40 days of age) were randomly allotted to four groups as a 2 × 2 factorial design: NT group, HT group, TRF group and HT coupled with TRF group (HT + TRF, *n* = 6 for each group). Each group had 3 replicates (2 pigs per replicate) and each of the replicates were raised in separate pens (3.0 m × 2.0 m). There were 3 separate rooms and 4 pens in each room were allotted evenly to the 4 groups. Pigs were subjected to a 7-d adaption and had free access to feed and water. Pigs in NT and TRF groups were maintained at 24 ± 3 °C throughout the trial, while HT and HT + TRF groups were exposed to a hot environment at 35 ± 2 °C from 11:00 to 15:00. During the heat exposure, portable heating instruments were put in the pens, and then pens were covered with polyurethane thermal insulation board on the top and side walls to maintain the temperature at 35 ± 2 °C. After heat exposure, the heating instruments and insulation boards were withdrawn to lower the temperature to that of NT group during the other time of the day. Pigs in NT and HT groups were fed three times a day at 6:00, 11:30, and 16:00 with a consume window of 10 h, while TRF groups were fed at 9:00, 11:30, and 14:00 to restrict the consume window in 5 h. Pigs usually spent 20 min to finish a meal and no restrictions on the provision of feedstuff during the consume window for all pigs. The remaining fodder in feeding trough that could not be accessed by pigs was checked and recorded at 20:00 for NT and HT groups and at 17:00 for TRF groups to determine the daily feed intake. All pigs were provided with standard commercial diets at stages from 15 to 40 kg of body weight, and the ingredient and nutrient values were shown in supplemental Table 1. The trial lasted for 61 days, from July 18th, 2021, to September 18th, 2021. BWs were monitored every 4–7 days, and average daily feed intake were obtained from three pens in each group. ADWG was calculated.

### Sample collection

After 12 h fasting, all pigs were slaughtered at a commercial slaughtering house according to the standard commercial procedures of the Chinese livestock production system. LTL muscle samples from the 10th to 13th rib at the left side of the carcass was removed within 45 min post-slaughter, and the samples were divided into two parts: the primary one was stored at 4 °C under vacuum for determination of meat quality, and the other was stored in -80 °C for detection of antioxidant status, amino acid and fatty acids composition.

### Carcass quality and chemical composition of LTL muscle determination

Carcass length was the length from the anterior edge of the pubic symphysis digitorum to the recess of the first cervical vertebra. Loin eye muscle area (LEMA) was the area of the cross-section of LTL muscle at the 10th rib, which was measured using sulfate paper and square measuring paper. The area was subsequently corrected for a body weight of 100 kg, and the formula was as follows (Friesen et al. 1994):

$$\text{Corrected LEMA }=\text{ Real LEMA }+ (100 -\text{ BWs}) \times \text{ Real LEMA }\div (\text{BWs }+ 70)$$

Muscle aliquots were weighted and placed in ventilated centrifugal tubes before being lyophilized. The weight difference between the initial and dried centrifugal tube was used to obtain the moisture content. Dried muscles were analyzed for crude protein and IMF content based on the Association of Official Agricultural Chemists (AOAC) (AOAC 2005).

### Meat quality measurement

Water-holding capacity was defined as a ratio of the retained moisture in the meat after a squeeze at a constant pressure of 350 N for 5 min (Wang et al. 2019). Briefly, meat was sliced at 1 cm thickness from the LTL muscle at 2 h post-mortem, and a round meat sample (the area is 5 cm^2^) was taken using a circular sampler. After weighted (W1), meat samples were sandwiched between two pieces of gauze, which were padded on the top and bottom with 18 pieces of filter paper each. Then the wrapped meat samples were put on the platform of the pressure gauge (Model Tenovo Meat-1, Beijing, China) and were pressurized to 350 N at a uniform speed. After 5 min holding at this pressure, meat samples were weighted (W2), and the WHC was calculated as:

$$\mathrm{WHC}\;(\%)=\lbrack\mathrm W1\times\mathrm A-(\mathrm W1-\mathrm W2)\rbrack\;/\;\mathrm W1\times\mathrm A\times100.\;\mathrm A\;\mathrm{is}\;\mathrm{the}\;\mathrm{ratio}\;\mathrm{of}\;\mathrm{moisture}$$

At 24 h post mortem, the meat color parameters, including lightness (L*), redness (a*) and yellowness (b*), were measured in triplicate from three sites of the meat by a CR400 Chroma Meter (Konica Minolta Sensing Inc., Osaka, Japan) according to the instruction of the manufacturer. The colorimeter had an illuminant D65, a 10° standard observer position, and an 8.0 mm diameter aperture. The blooming time was 1 s. Three values of each sample were averaged for each parameters.

At 24 h post mortem, drip loss of the LTL muscle was measured in a method reported by the previous study (Young et al. 2004). Three strip-shaped LTL muscles (about 25 g for each replicate) were removed from each sample, weighted (W1) and hung in a nitrogen-filled container at 4 °C for 24 h, avoiding contact with the container. Subsequently, the replicates were wiped with blotting paper and weighted again (W2). The drip loss$(\%)\;=(\mathrm W1-\mathrm W2)\;/\;\mathrm W1\times100$. Mean value of the three replicates was the drip loss of each sample.

Immediately after the determination of drip loss, meat samples were packed in polyethylene bags with water that was just enough to submerge the meat, and then bags were cooked in a water bath at 75 °C for 15 min to reach an internal temperature of 70 °C, based on the method in the previous study (Cong et al. 2017). The cooked meat were cooled to room temperature on the absorbent paper, and surface moisture was wiped before being reweighted (W3).

The cooking loss $(\%)=\;(\mathrm W2-\mathrm W3)\;/\;\mathrm W2\;\times\;100$. Mean value of the three replicates was the cooking loss of each sample.  

The three cooked strip-shaped LTL muscles were individually cut into six strips (1 cm × 1 cm × 3 cm) for each sample, parallel to the muscle fiber, and were used to determine the shear force. Each strip was measured in duplicate with a Digital Meat Tenderness Tester (Guangzhou Runhu Instruments Co., Ltd, Guangzhou, China) according to the operation instructions.

### Intramuscular components and metabolic enzyme activities

Glycogen, lactic acid, and hydroxyproline contents and metabolic enzyme activities of succinic dehydrogena se, malate dehydrogenase, and lactate dehydrogenase in LTL muscle were measured using commercial kits from Nanjing Jiancheng Bioengineering Institute following the manufacturer's protocol.

### Meat oxidative status

Meat samples were suspended in normal saline to homogenize. Subsequently, the homogenates were centrifuged and the supernatant was collected for protein concentration determination using a commercial BCA protein assay kit (Scientific Phygene, Fuzhou, China).

The content of MDA and enzyme activities of GSH-Px, CAT, and T-SOD were measured using commercial kits from Nanjing Jiancheng Bioengineering Institute according to the manufacturer's protocol.

### The amino acid composition of LTL muscle

The measurement was in accordance with the national standard for food safety–determination of amino acids in food (GB 5009.124–2016) issued by China's food and drug administration. Briefly, about 1 g chopped LTL sample was weighed in a grass test tube and mixed with 15 ml 6 M hydrochloric acid. After vacuuming and filling with nitrogen, the tubes with mixtures were hydrolyzed at 110 °C for 22–24 h. Subsequently, mixtures were filtered into a 50 ml volumetric flask and diluted to calibration tail with ultrapure water. Then 1 ml mixture was transferred to a 15 ml test tube and dried at 45 °C with a multivapor, and the dry matter was dissolved with 1.5 ml sodium citrate buffer with a pH of 2.2. The solution was filtered using a 0.22 μm membrane filter into an auto-sampler vial before amino acid analysis with an L-8900 amino acid analyzer (HITACHI, Tokoyo, Japan).

### The fatty acids composition of LTL muscle

The FA composition was determined according to the national standard for food safety determination (GB 5009.168–2016) with a slight modification. Firstly, lipid in 5 g LTL muscle samples was extracted by the method described in the previous report (Li et al. 2017). The extraction was then mixed with 8 ml 2% NaOH methanol solution in a flask connected to a reflux condenser and heated in a water bath at 80 °C until all the mixture evaporated. Then 7 ml 15% BF_3_-methanol solution was added from the upper end of the reflux condenser, and the mixture was heated in a water bath at 80 °C for another 2 min. After cooling down to room temperature, 10 ml n-hexane and 20 ml saturated NaCl solution were added to the mixture, which was shaken for 2 min and settled down for stratification. About 5 ml n-heptane extraction solution was obtained and transferred into a 25 mL test tube, which was added with 5 g sodium sulphate anhydrous and shaken for 1 min and stewed for 5 min. The fatty acids methyl esters were separated and determined by gas chromatography (Agilent Technologies, CA, US) using a 100 m × 0.25 mm capillary column and 0.25 μm film thickness (Agilent Technologies, CA, US). The injector and detector temperatures were held at 280 °C and 270 °C, respectively. The column temperature profiles were set as follows: the initial was held at 100 °C for 13 min; increased to 180 °C at 10 °C/min and held for 6 min; increased to 200 °C at 1 °C/min and held for 20 min; increased to 230 °C at 4 °C/min and held for 10.5 min. Fatty acids were finally determined using a flame ionization detector, and chromatograms were integrated using GC Chemstation software (Agilent Technologies, CA, US), and FAME peaks were identified using the 37 FAME Mix (Supelco, PA, US).

### RNA extraction and qRT-PCR

Total RNA was extracted from the muscle by the TRIzol agent (Invitrogen, CA, US) and the concentration and purity were measured spectrophotometrically at 260 and 280 nm with Nanodrop 8000 (Thermo Fisher Scientific, Wilmington, US). Then the RNA samples were reverse-transcribed to cDNA with the M-MLV reverse transcriptase (TaKaRa, Dalian, China). The 20 μl the reaction system, composed of 14 μl double distilled water, 4 μl reverse transcription reagent and 2 μl RNA samples, were heated to 37 °C and held for 15 min, then heated to 85 °C and held for 5 s, and finally kept at 4 °C. The housekeeping gene glyceraldehyde-3-phosphate dehydrogenase (GAPDH) and target genes, including *MYH7*, *MYH2*, *MYH1, MYH4*, *MyOG*, *MyOD*, *MAFbx*, *MuRF1*, *Nrf2*, *NQO1*, *GCLC*, *HO-1*, *FADS1*, *FADS2*, *Elovl2* and *Elovl5* were quantified by real-time PCR on a QuantStudioTM 3 system using a commercial kit (SYBR Premix Ex Taq, TaKaRa, Dalian, China). The gene-specific primers were designed based on the corresponding mRNA sequences with Primer Version 5.0 (Table S2). Each sample was measured in triplicate. Ct values were normalized to the GAPDH gene and a relative mRNA transcript level was achieved by the 2^−ΔΔCt^ method. These normalized values would determine the degree of induction or inhibition presented as a "fold difference" compared to the values in NT group.

### Proteins extraction and western blot

Proteins were extracted by a commercial kit (Beyotime Institute of Biotechnology, Nantong, China) and the concentrations were measured with the BCA Protein Assay Protocol. Then quantifications of MyHC7 (proteintech, 22,280–1-AP, 1:1000, rabbit antibody), MYO (proteintech, 66,205–1-Ig, 1:1000, mouse antibody), Nrf2 (ABclonal, A0674, 1:1000, rabbit antibody), HO-1 (proteintech, 10,701–1-AP, 1:1000, rabbit antibody), HSP70 (Bioss, bs-0244R, 1:1000, rabbit antibody), FADS1 (proteintech, 10,627–1-AP, 1:1000, rabbit antibody), FADS2 (ABclonal, A10270, 1:1000, rabbit antibody) and β-actin (proteintech, 66,009–1-Ig, 1:2000, mouse antibody) were conducted by Western blot analysis. Each of the samples (40 μg) was loaded in separate lane of 10% PAGE with a MiniProtean Tetra System (BioRad, CA, US) according to the Precision Plus Protein molecular weight standards (BioRad). Then proteins in PAGE were transferred to nitrocellulose membranes (EMD Millipore Corporation, MA, US), which were incubated with 5% non-fat milk solution for 1 h at room temperature to prevent non-specific binding. Membranes were incubated with primary antibodies overnight at 4 °C, and then washed and incubated with HRP-labeled goat anti-rabbit IgG (Huaxingbio, HX2031, 1:2000) or anti-mouse IgG (Huaxingbio, HX2032 1:2000) based on the source of the primary antibody. Membrane visualization was performed using an enhanced chemiluminescence detection reagents. Bands were scanned and the relative intensities were measured by densitometry using Scion Image v. 4.0.2 (Scion Corporation, Frederick, US). β-actin was regarded as the cytosolic control.

### Statistical analysis

Data were analysed in a completely randomized design using the GLM procedure using IBM SPSS 19.0 statistics for Windows (IBM Inc., NY, USA). The mixed model was composed of the fixed effects of HT, TRF, their interactions and the random effect of pen, which was adopted as follows:

$${Y}_{\text{ijkl}} = \mu + {T}_{\text{i}} + {F}_{\text{j}} + {P}_{k}(F) + {\left(T \times F\right)}_{\text{ij}} + {e}_{\text{ijkl}}$$

where *Y*_ijkl_ is the observation, *μ* is the overall mean, *T*_i_ is the fixed effect of temperature (i = NT; HT), *F*_j_ is the fixed effect of feeding regime (j = non-TRF; TRF), *P*_k_(*F*) is the random effect of the pen nested with the feeding regime, (*T* × *F*)_ij_ is the interaction between temperature and feeding regime, and *e*_ijkl_ is the residual error. Post hoc testing was employed using Duncan multiple comparison tests of the means. Statistical difference was declared at *P* < 0.05.

## Supplementary Information

Supplementary Material 1: Supplementary table 1 Ingredients (% as-fed basis unless otherwise indicated) and nutrient values of diets ^1^The premix provided the following per kg of diets: VA 9000 IU, VD_3_ 1500 IU, VE 40 IU, VK_3_ 1.5 mg, VB_1_ 4.5 mg, VB_2_8 mg, VB_6_ 6 mg, VB_12_ 60μg, nicotinic acid 20 mg, pantothenic acid 30 mg， folic acid 0.8 mg, biotin 0.24 mg, Fe (as ferrous sulfate) 200 mg, Zn (as zinc sulfate) 200 mg, Cu (as copper sulfate) 100 mg, Mn (as manganese sulfate) 50 mg, I (as potassium iodide) 0.6 mg, Se (as sodium selenite) 0.3 mg. Supplementary table 2 Primers sequences used for real-time quantitative PCR analysis. Supplementary table 3 Effects of heat exposure and time-restricted feeding on metabolic enzyme activities in *longissimus thoracis et lumborum* muscle Means within a row with different superscript letters are significantly different (*P *< 0.05). The results were presented as mean values with SED (standard error of difference, *n* = 6).^1^ LDH, lactate dehydrogenase. MDH, malate dehydrogenase. SDH, succinic dehydrogenase.^2^NT, thermal neutral. HT, heat treatment. TRF, time-restricted feeding. Supplementary figure 1 Effects of heat exposure and time-restricted feeding on body weight (A) and daily feed intake (B) monitored in certain days during the whole trial. NT, thermal neutral. HT, heat treatment. TRF, time-restricted feeding. HT+TRF, co-treatment of HT and TRF.

## Data Availability

The data and materials that support the findings of this study are available from the corresponding author upon request.

## Abbreviations

TRF: Time-restricted feeding
HT: Heat treatment
NT: Thermal neutral
LTL: Longissimus thoracis et lumborum
PUFAs: Polyunsaturated fatty acids
SFAs: Saturated fatty acids
L^*^: Lightness
a^*^: Redness
b^*^: Yellowness
PSE: Pale, soft and exudative
IMF: Intramuscular fat
BWs: Body weights
ADFI: Average daily feed intake
ADWG: Average daily weight gain
WHC: Water holding capacity
GSH-Px: Glutathione peroxidase
MDA: Malondialdehyde
CAT: Catalase
T-SOD: Total superoxide dismutase
SDH: Succinic dehydrogenase
MDH: Malate dehydrogenase
LDH: Lactate dehydrogenase
Nrf2: Nuclear factor (erythroid-derived 2)-like 2
HO-1: Heme oxygenase-1
NQO1: NAD (P)H/quinone oxidoreductase 1
GCLC: Glutamate-cysteine ligase catalytic subunit
HSP70: Heat shock protein 70
MYH7: Myosin heavy chain7
MyOG: Muscle growth-related genes myogenic
MyOD: Myogenic differentiation
MuRF1: Muscle-specific RING-finger protein 1
MAFbx: Atrophy-related genes atrogen-1
EAA: Essential amino acids
NEAA: Nonessential amino acids
Met: Methionine
Asp: Asparagine
Cys: Cysteine
FADS1: Fatty acid desaturase 1
FADS2: Fatty acid desaturase 2
Elovl2: Elongation-of-very-long-chain-fatty acids 2
Elovl5: Elongation-of-very-long-chain-fatty acids 5
