# Supplementary material for: Time-restricted feeding relieves high temperature-induced impairment on meat quality by activating the Nrf2/HO-1 pathway, modification of muscle fiber composition, and enriching the polyunsaturated fatty acids in pigs
Source: Stress Biol. 2024 Sep 14;4(1):39. doi: 10.1007/s44154-024-00182-w (PMC11401797; doi:10.1007/s44154-024-00182-w)
Supplement: Supplementary file 1 — Supplementary Material 1: Supplementary table 1 Ingredients (% as-fed basis unless otherwise indicated) and nutrient values of diets 1The premix provided the following per kg of diets: VA 9000 IU, VD3 1500 IU, VE 40 IU, VK3 1.5 mg, VB1 4.5 mg, VB28 mg, VB6 6 mg, VB12 60μg, nicotinic acid 20 mg, pantothenic acid 30 mg， folic acid 0.8 mg, biotin 0.24 mg, Fe (as ferrous sulfate) 200 mg, Zn (as zinc sulfate) 200 mg, Cu (as copper sulfate) 100 mg, Mn (as manganese sulfate) 50 mg, I (as potassium iodide) 0.6 mg, Se (as sodium selenite) 0.3 mg. Supplementary table 2 Primers sequences used for real-time quantitative PCR analysis. Supplementary table 3 Effects of heat exposure and time-restricted feeding on metabolic enzyme activities in longissimus thoracis et lumborum muscle Means within a row with different superscript letters are significantly different (P < 0.05). The results were presented as mean values with SED (standard error of difference, n = 6).1 LDH, lactate dehydrogenase. MDH, malate dehydrogenase. SDH, succinic dehydrogenase.2NT, thermal neutral. HT, heat treatment. TRF, time-restricted feeding. Supplementary figure 1 Effects of heat exposure and time-restricted feeding on body weight (A) and daily feed intake (B) monitored in certain days during the whole trial. NT, thermal neutral. HT, heat treatment. TRF, time-restricted feeding. HT+TRF, co-treatment of HT and TRF. [file 44154_2024_182_MOESM1_ESM.docx]

Supplementary table 1

| Items | Content (%) |
| --- | --- |
| Corn | 64.60 |
| Wheat bran | 17.00 |
| Soybean meal | 13.50 |
| Soybean oil | 2.00 |
| Limestone | 0.96 |
| CaHPO_4_ | 0.42 |
| NaCl | 0.30 |
| L-Lysine·HCl | 0.20 |
| DL-Met | 0.02 |
| Premix^1^ | 1.00 |
| Total | 100 |
| Calculated nutrient levels | |
| Digestive energy (MJ/kg) | 13.49 |
| Crude protein | 15.03 |
| Crude fiber | 2.91 |
| Calcium | 0.54 |
| Total phosphorus | 0.50 |
| Lysine | 0.84 |
| Methionine | 0.25 |
| Fatty acids (g/100 g of total fatty acids) | |
| C12:0, lauric | 0.06 |
| C14:0, myristic | 0.15 |
| C16:0, palmitic | 19.44 |
| C16:1, palmitoleic | 0.23 |
| C18:0, stearic | 3.35 |
| C18:1 n-9, oleic | 19.57 |
| C18:2 n-6, linoleic | 48.76 |
| C18:3 n-3, linolenic | 3.41 |
| C20:0, arachidic | 0.08 |

Supplementary table 2

| Gene | Genbank number | Primer sequence (5’ to 3’) | Product size (bp) |
| --- | --- | --- | --- |
| GAPDH | NM_001206359.1 | F: ACACTGAGGACCAGGTTGTG | 98 |
|  |  | R: GACGAAGTGGTCGTTGAGGG |  |
| MYH7 | NM_213855.2 | F: CGTGGACTACAACATCATAGGC | 146 |
|  |  | R: CCTTCTCAACAGGTGTGTCG |  |
| MYH2 | NM_214136.1 | F: CATTGAGGCCCAGAATAGGC | 127 |
|  |  | R: TGCTTCCGTCTTCACTGTCAC |  |
| MYH1 | NM_001123141.1 | F: AGTGCTATCCCAGAGGGTCAGT | 161 |
|  |  | R: AGCTTTTCATCTCGCATCTCCT |  |
| MYH4 | NM_001104951.2 | F: TTGACTGGGCTGCCATCAAT | 111 |
|  |  | R: GCCTCAATGCGCTCCTTTTC |  |
| MyoD | NM_001002824.1 | F: AAGTCAACGAGGCCTTCGAG | 279 |
|  |  | R: GGGGGCCGCTATAATCCATC |  |
| MyoG | NM_001012406.1 | F: GAGCTGTATGAGACATCCCCC | 75 |
|  |  | R: GTGGACGGGCAGGTAGTTTT |  |
| MAFbx | NM_001044588.1 | F: GAGAAGAGTGGCAGCTTCGT | 111 |
|  |  | R: TCTCTTCTTGGCCGCAACAT |  |
| MuRF1 | NM_001184756.1 | F: AGCACGAAGACGAGAAAATC | 150 |
|  |  | R: TGCGGTTACTCAGCTCAGTC |  |
| Nrf2 | XM_013984303.2 | F: AGCGGATTGCTCGTAGACAG | 155 |
|  |  | R: TCAATCAAATCCATGTCCTTGGC |  |
| HO-1 | NM_001004027.1 | F: TACCGCTCCCGAATGAACAC | 209 |
|  |  | R: GTCACGGGAGTGGAGTCTTG |  |
| NQO1 | NM_001159613.1 | F: CATGGCGGTCAGAAAAGCAC | 135 |
|  |  | R: ATGGCATACAGGTCCGACAC |  |
| GCLC | XM_021098556.1 | F: GGACAAACCCAAACCATC | 133 |
|  |  | R: CGGCGTTTCCTCATATTG |  |
| FADS1 | NM_001113041.1 | F: GTCACTGCCTGGCTCATTCT | 155 |
|  |  | R: AGGTGGTTCCACGTAGAGGT |  |
| FADS2 | NM_001171750.1 | F: TCATGGAGATTGACCGCGAG | 226 |
|  |  | R: AGCGGCTTTTCCTGGTACTC |  |
| Elovl2 | XM_021100182.1 | F: CCTTAAGTCTCTGCGGGCAA | 133 |
|  |  | R: ACCACCCTCTGACTCGAGAA |  |
| Elovl5 | XM_021098832.1 | F: TACCACCATGCCACTATGCT | 102 |
|  |  | R: GACGTGGATGAAGCTGTTGA |  |

Supplementary table 3

| Item^1^ | -TRF^2^ | | +TRF | | SED | Main effect, HT | | Main effect, TRF | | *P*-value | | |
| --- | --- | --- | --- | --- | --- | --- | --- | --- | --- | --- | --- | --- |
|  | NT | HT | NT | HT |  | NT | HT | -TRF | +TRF | HT | TRF | HT × TRF |
| LDH (U/mgprot) | 1.08 | 1.15 | 1.15 | 1.27 | 0.08 | 1.16 | 1.21 | 1.11 | 1.21 | 0.29 | 0.29 | 0.80 |
| MDH (U/mgprot) | 0.83 | 1.21 | 0.90 | 0.82 | 0.10 | 0.86 | 1.01 | 1.02 | 0.86 | 0.17 | 0.15 | 0.04 |
| SDH (U/mgprot) | 1.55 | 1.81 | 1.76 | 1.53 | 0.18 | 1.65 | 1.67 | 1.68 | 1.65 | 0.92 | 0.85 | 0.21 |


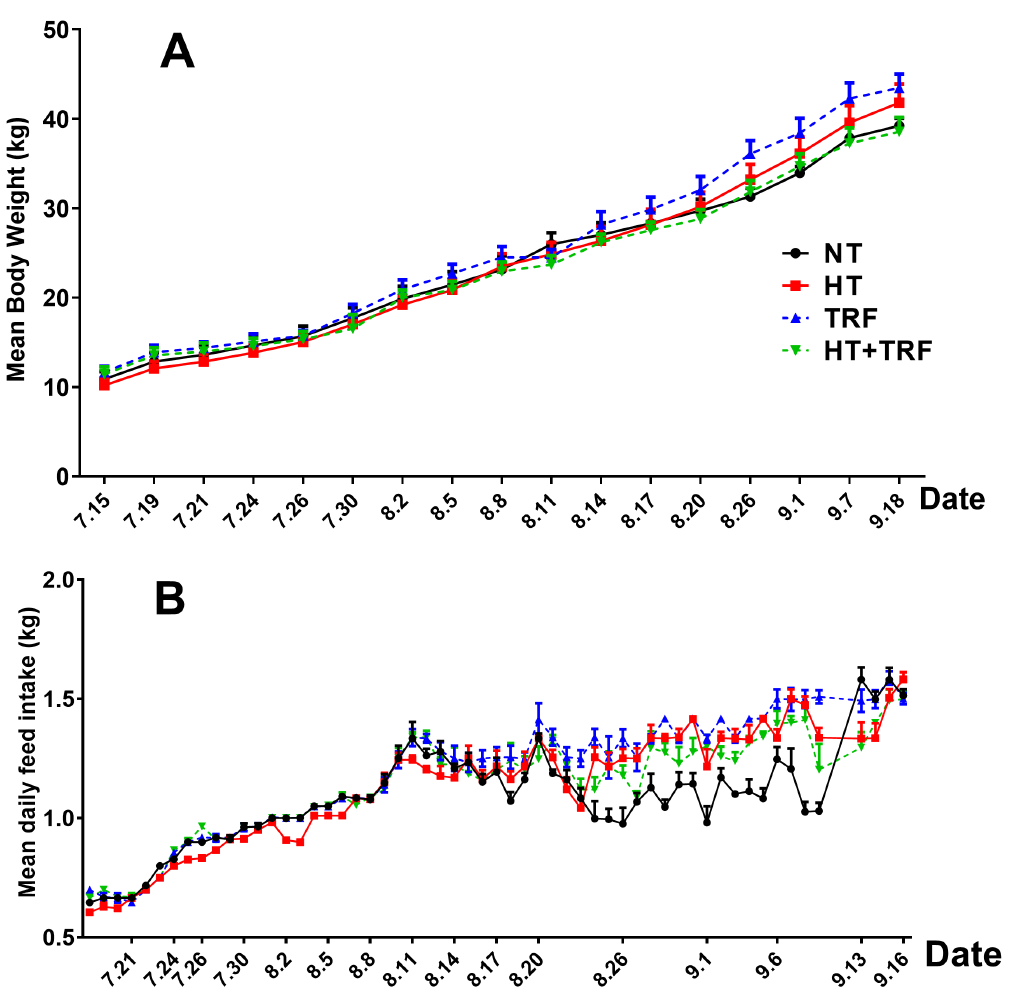


Supplementary figure 1
